# Fatigue Strength Estimation Based on Local Mechanical Properties for Aluminum Alloy FSW Joints

**DOI:** 10.3390/ma10020186

**Published:** 2017-02-15

**Authors:** Kittima Sillapasa, Yoshiharu Mutoh, Yukio Miyashita, Nobushiro Seo

**Affiliations:** 1 Industrial Engineering Department, Ubon Ratchathani University, Ubon Ratchathani 34190, Thailand; 2 Professor Emeritus, Nagaoka University of Technology, Nagaoka-shi 940-2188, Japan; mutohyoshiharu@yahoo.co.jp; 3 Mechanical Engineering Department, Nagaoka University of Technology, Nagaoka-shi 940-2188, Japan; miyayuki@mech.nagaokaut.ac.jp; 4 Nippon Light Metal Ltd., Shizuoka-shi 421-3203, Japan; nobushiro-seo@nikkeikin.co.jp

**Keywords:** fatigue strength estimation, local fatigue strength, hardness, friction stir welding, aluminum alloy

## Abstract

Overall fatigue strengths and hardness distributions of the aluminum alloy similar and dissimilar friction stir welding (FSW) joints were determined. The local fatigue strengths as well as local tensile strengths were also obtained by using small round bar specimens extracted from specific locations, such as the stir zone, heat affected zone, and base metal. It was found from the results that fatigue fracture of the FSW joint plate specimen occurred at the location of the lowest local fatigue strength as well as the lowest hardness, regardless of microstructural evolution. To estimate the fatigue strengths of aluminum alloy FSW joints from the hardness measurements, the relationship between fatigue strength and hardness for aluminum alloys was investigated based on the present experimental results and the available wide range of data from the references. It was found as: *σ_a_* (*R* = −1) = 1.68 HV (*σ_a_* is in MPa and HV has no unit). It was also confirmed that the estimated fatigue strengths were in good agreement with the experimental results for aluminum alloy FSW joints.

## 1. Introduction

From both environmental and energy considerations, the demand for light weight structures and machines are increasing. The application of friction stir welding is one of the promising ways to enhance the introduction of light materials into structural components.

There are normally four regions in a friction stir welding (FSW) joint: the base metal (BM), the heat affected zone (HAZ), the thermo-mechanically affected zone (TMAZ), and the stir zone or dynamically recrystallized zone (SZ or DRZ) [1,2,3,4,5,6]. Microstructure, hardness, and residual stress are varied throughout these regions. The SZ shows low hardness compared to the BM for heat-treatable materials [2,3,5,6]. In structural applications of the joint, fatigue characteristics is one of the main concerns. The fatigue strengths of FSW joints, where fatigue failure often nucleates at the HAZ/TMAZ, tend to be lower than those of corresponding base materials but much higher than those of traditional fusion welded joints [2,3,4,7,8,9].

For the fatigue tests of dissimilar FSW butt-joints for aluminum alloys, there have been a limited number of reports: Cavaliere et al. [10] reported that the higher fatigue strength of the 6082–2024 dissimilar FSW joint can be obtained when softer AA2024 aluminum alloy is used in the retreating side. They [11] also reported that the highest fatigue strength of the 2024–7075 dissimilar FSW joint can be obtained when the tool position is in the 2024 side at a distance of 1 mm from the weld interface. Details of the fatigue behavior of dissimilar FSW joints, such as the relationship between the fatigue strength of each location (such as SZ, HAZ, and BM) and the overall fatigue strength of the dissimilar FSW joint and the relationship between the hardness distribution and the overall fatigue strength of the dissimilar FSW joint, have not yet been clarified. Therefore, a convenient cost-performance fatigue strength estimation method for aluminum alloy FSW joints has also not yet been developed.

In the previous work [12], as the first step towards the study of fatigue behavior and a fatigue strength estimation method of aluminum alloy FSW joints, the tensile strength and fatigue strength characteristics of the 6N01 similar FSW joints were investigated in detail. The local tensile strength and local fatigue strength were also investigated by using small round bar specimens extracted from their respective locations: the lowest tensile strength and fatigue strength were found in the HAZ. Overall fatigue strength of the similar FSW joint corresponded to the lowest fatigue strength of the small specimen extracted from the HAZ.

In the present study, as the next step of this series of studies, tensile strength and fatigue strength tests of the 6N01-7N01 dissimilar FSW joint and the 7N01 similar FSW joint were carried out. Fatigue characteristics of these FSW joints were investigated in detail by combining the local tensile strength and local fatigue strength obtained by using small round bar specimens extracted from the respective locations of the joint. Based on the results, a convenient cost-performance fatigue strength estimation method based on local mechanical properties for the aluminum alloy FSW joints has been proposed.

## 2. Experimental Procedure

### 2.1. Materials

The materials used in the present study were 6N01 and 7N01 aluminum alloy plates with a thickness of 6 mm, a width of 160 mm, and a length of 500 mm, the chemical compositions of which are shown in Table 1. The heat-treatment condition of both plates was T5.

### 2.2. Friction Stir Welding

**Figure 1:**
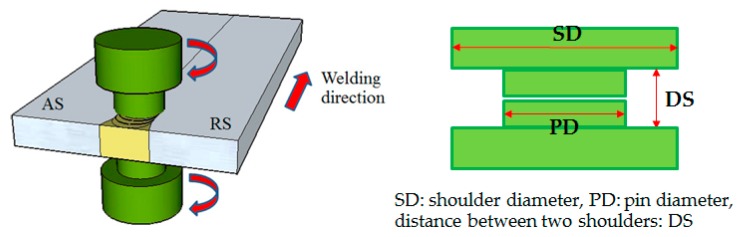
Schematics of the friction stir welding (FSW).

Dissimilar friction stir welding (FSW) between the 6N01 and 7N01 plates as well as similar FSW of the 7N01 plate was conducted by using a conventional machining center with the same tool as in the previous study [12], which was a bobbin type tool with shoulder diameter (SD) of 20 mm, pin diameter (PD) of 12 mm, and distance between the two shoulders (DS) of 5.8 mm, as schematically shown in Figure 1. The received starting plates with a width of 160 mm were cut by a metal cutting saw to obtain FSW welding plates with a width of 80 mm. FSW welding was conducted on an as-cut end-surface without any surface finish, such as grinding or polishing. The dissimilar FSW joint between 6N01 and 7N01 and the similar FSW joint of 7N01 are named hereafter as the 6N01-7N01 FSW and the 7N01 FSW, respectively. The rotation speed and welding speed were determined based on the pre-trial joining to find the best welding condition without flash and flaws. The resultant rotation and welding speeds were 300 rpm and 200 mm/min for the 7N01 FSW and 400 rpm and 300 mm/min for the 6N01-7N01 FSW, while those for the 6N01 similar FSW joint (hereafter 6N01 FSW) were 500 rpm and 400 mm/min, as reported in the previous paper [12]. Therefore, the welding condition for the 6N01-7N01 FSW was in the middle of those for the two similar FSW joints of 6N01 and 7N01.

### 2.3. Microstructural Observation, Micro-Hardness Test, and Residual Stress Measurement

Microstructural observation and micro-hardness measurement of the 6N01-7N01 FSW and the 7N01 FSW were carried out on the cross-section obtained by transversely cutting the joint. The microstructural observation was conducted by using an optical microscope (Nikon: Eclipse LV150, Nikon Co., Tokyo, Japan). The micro-hardness tests were conducted by using a micro hardness tester (Shimadzu HMV, Shimadzu Co., Kyoto, Japan) with a load of 5 N for 10 s. The residual stress in the longitudinal direction of the specimen, which was the traverse direction to the FSW direction, was measured by a conventional XRD machine (Shimadzu XRD-6100, Shimadzu Co., Kyoto, Japan) under the same measuring conditions as in the previous paper [12].

### 2.4. Tensile Test

**Figure 2:**
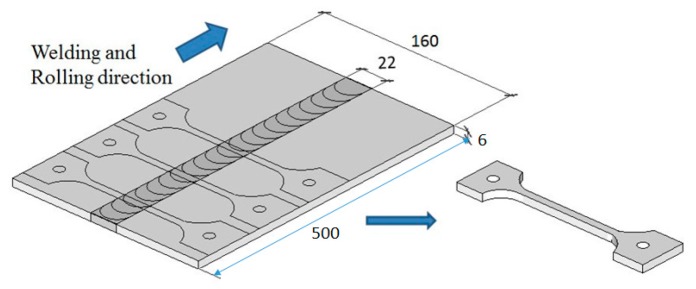
Subtraction of the plate specimen.

**Figure 3:**
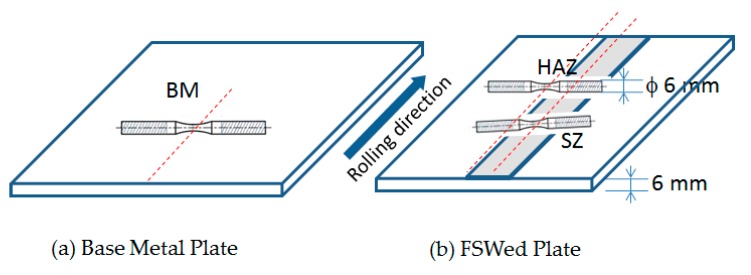
Subtraction of the small round bar specimen: BM: Base metal, SZ: Stir zone at the center of joint, HAZ: Heat affected zone with the lowest hardness.

**Figure 4:**
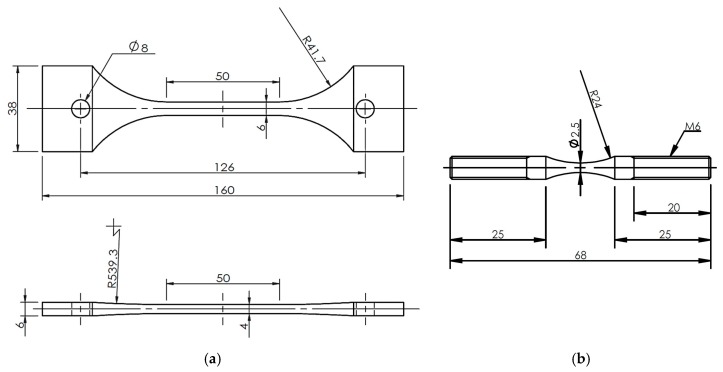
Geometries and dimensions of the plate and round bar specimens. (**a**) Plate specimen; (**b**) Small round bar specimen.

Two types of tensile specimens were prepared: one was the plate specimen and the other was the small round bar specimen. The plate specimens were cut from the FSW joint, as shown in Figure 2, where the whole FSW joining region was included in the gage part of the specimen. The small round bar specimens were cut from specific locations, such as the base metal (BM), the heat affected zone (HAZ), and the stir zone (SZ), as shown in Figure 3, to investigate the local tensile strength at each location. The geometries and dimensions of the two types of specimens are shown in Figure 4. The stress concentration of the present hour-glass shape specimen was 1.03, which was small enough, and no notch effect on the test result was assumed. Tensile tests were conducted on an Instron-type tensile test machine (Shimadzu DSS-10T-S, Shimadzu Co., Kyoto, Japan) under a displacement rate of 1 mm/min. Before the tensile tests, the specimen surfaces were polished with emery papers up to #1000. At least three specimens were tested for each condition to obtain the average tensile strength. The period spent from FSW joining until specimen preparation before the test was more than one month.

### 2.5. Fatigue Test

Fatigue tests were carried out on a servo-hydraulic fatigue test machine (Shimadzu EHF-LV010K1-A10, Shimadzu Co., Kyoto, Japan) with a stress ratio of 0.1 at a frequency of 20 Hz. The same two kinds of specimens used for the tensile tests were also used for the fatigue tests. The specimen surfaces were polished under the same conditions as for the tensile test specimens. The plate specimens were connected with the fatigue test jigs with holes of 10 mm diameter by inserting the round bar pins with a diameter of 7 mm into the holes for applying fatigue load. The round bar specimens with screw thread ends were fixed to the fatigue test jig with internal screw threads by using clamping nuts. Four or five specimens were tested at different stress amplitude levels to obtain the S-N curve.

## 3. Experimental Results

### 3.1. Microstructure and Micro-Hardness

**Figure 5:**
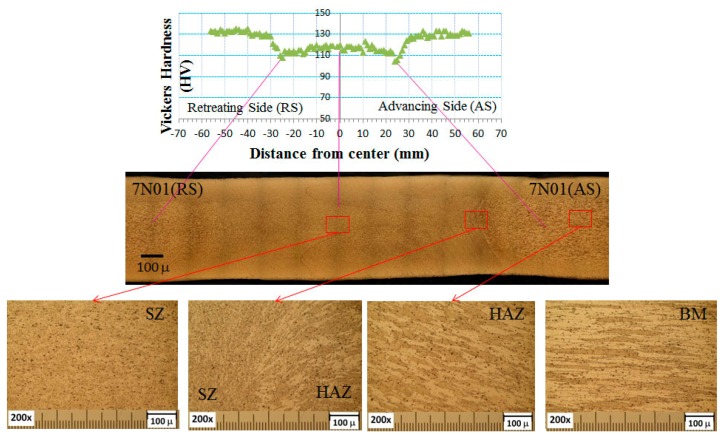
Microstructure and hardness distribution for the 7N01 friction stir welding (FSW).

The results of the microstructural observation and micro-hardness measurements of the 7N01 FSW are shown in Figure 5. As seen from the figure, the joint can be divided into three regions: BM, HAZ, and SZ. The elongated large grains with the highest hardness were found in BM. The similar elongated large grains as in the BM but with the lowest hardness were observed in the HAZ, while the recrystallized fine grains with intermediate hardness were found in the SZ. The qualitative characteristics of these three regions were almost similar to those found in the 6N01 FSW [12].

**Figure 6:**
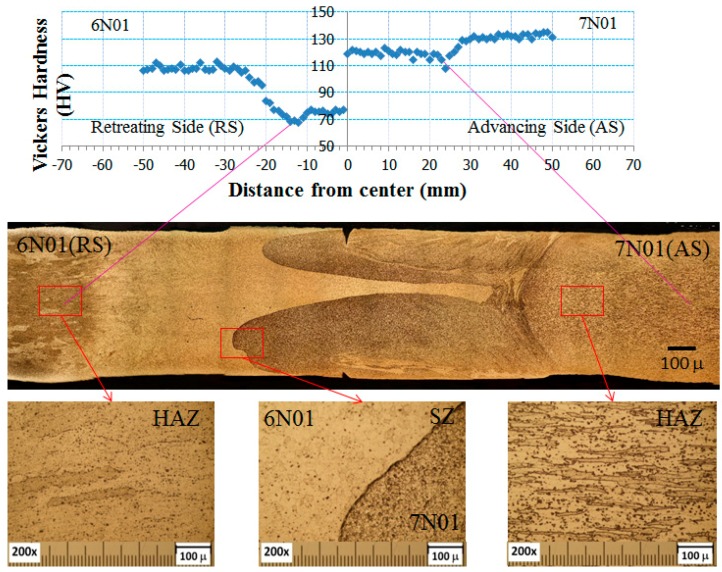
Microstructure and hardness distribution for the 6N01-7N01 FSW.

**Figure 7:**
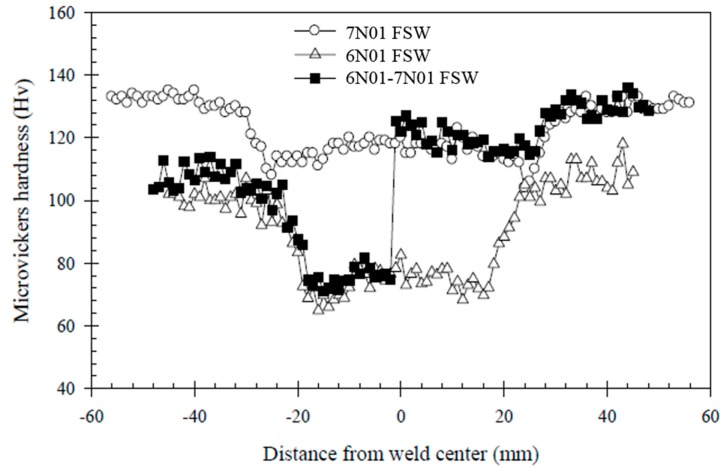
Hardness distributions for the three FSW joints.

The results of microstructural observation and micro-hardness measurements of the 6N01-7N01 FSW are shown in Figure 6. As seen from the figure, the joint can be divided into six regions: BM, HAZ, and SZ in the 7N01 side and SZ, HAZ, and BM in the 6N01 side. In the center of the joint, both the 6N01 and 7N01 were recrystallized but the two materials were isolated and not homogenized due to the solid state stirring process of the FSW. The similar solid state stirring morphology was also reported in the dissimilar FSW joints [13,14]. For comparison purposes, the hardness distributions for the 6N01 FSW and 7N01 FSW and that for the 6N01-7N01 FSW are shown in Figure 7. As seen from the figure, the hardness distributions in the 6N01 side and the 7N01 side of the 6N01-7N01 FSW almost coincide with those of the 6N01 FSW and the 7N01 FSW, respectively. According to the above detailed microstructural observations, no defects and pores in the FSW region could be observed due to the selection of suitable FSW conditions, as seen in Figure 5 and Figure 6.

### 3.2. Residual Stress

**Figure 8:**
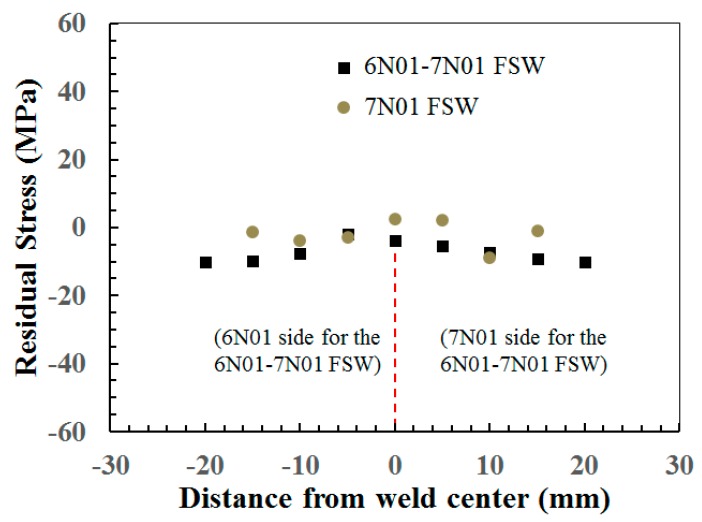
Residual stress distribution for the 7N01 FSW and the 6N01-7N01 FSW.

From the residual stress measurements, the transverse residual stress distributions, which would influence the fatigue strength in the fatigue test with an applied load perpendicular to the welding direction, for the 7N01 FSW and the 6N01-7N01 FSW are shown in Figure 8. The only negligible level of residual stress was found from the figure for both the joints, which was also similar to that observed in the 6N01 FSW [12]. Small transverse residual stress compared to the longitudinal residual stress was often reported [10,13]. Small residual stress (20–30 MPa) for the 7050-T7451–2024-T351 dissimilar FSW joint [15] and that (less than 80 MPa) for the 2024-T3–6082-T6 dissimilar FSW joint [16] were also reported. A similar small residual stress in the FSW joint compared to that in the tungsten inert gas (TIG) welding with melting process was also reported [17]. As one of the reasons for the small residual stress induced in the FSW joint, it is speculated that the FSW has no shrinkage process in the melting region, which induces high residual stress in conventional welding. Furthermore, the recrystallization in the stir zone may contribute to reducing the residual stress in the FSW joint. Another possible reason for significantly small residual stress in the present FSW joint will be due to the butt welding of plates with 6 mm thickness, not of structure components with constraints due to rigid geometry.

### 3.3. Tensile Strength

**Figure 9:**
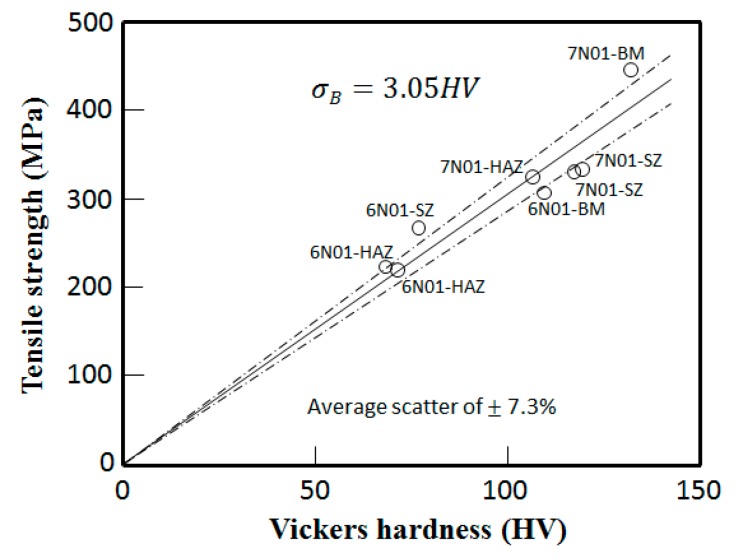
Relationship between the Vickers hardness and tensile strength for various locations in the 6N01 FSW and the 7N01 FSW.

The results of the tensile tests are summarized in Table 2, where the tensile strengths indicated are the average values of three samples. The results for the 6N01 FSW are referred from the previous study [12]. For the plate specimens, the tensile fracture occurred in the HAZ region for the 6N01 FSW and 7N01 FSW and also in the HAZ region of the 6N01 side for the 6N01-7N01 FSW. Considering the tensile strengths in BM, SZ, and HAZ obtained by the small round bar specimens, it is suggested that tensile fracture of the FSW joint would occur at the lowest tensile strength location, which is also at the lowest hardness location. The relationship between the Vickers hardness and tensile strength for the present aluminum alloy FSW joints is shown in Figure 9, where the tensile strengths are those of round bar specimens listed in Table 2 and the Vickers hardness values are the average hardness values in each zone obtained from the hardness distributions shown in Figure 7. Since a linear relationship between hardness and tensile strength with some scatter is well known in steels [18,19,20], a similar linear relationship will be expected even in softer materials such as aluminum alloys. For the present FSW joints, the relationship can be expressed as:

(1) *σ_B_* = 3.05 HV

The average percentage of scatter S calculated by Equation (2)

(2) $$S=\frac{1}{n}\overset{n}{\underset{i=1}{\sum }}\left(\frac{\sigma_{B}^{est}-\sigma_{B}^{exp}}{\sigma_{B}^{exp}}\times 100\right)i$$

was 7.3% as indicated in Figure 9, where $\sigma_{B}^{est}$ is the tensile strength estimated by Equation (1) and $\sigma_{B}^{exp}$ is the experimental results of the tensile tests. Similar relationships have been reported: *σ_B_* = 3.0–3.5 HV for aluminum alloys [21] and *σ_B_* = 3.12 HV for ductile materials [22]. Based on this relationship, by replacing tensile strength with hardness, it can be said that tensile fracture of the FSW joint will occur at the lowest hardness location.

### 3.4. Fatigue Strength

**Figure 10:**
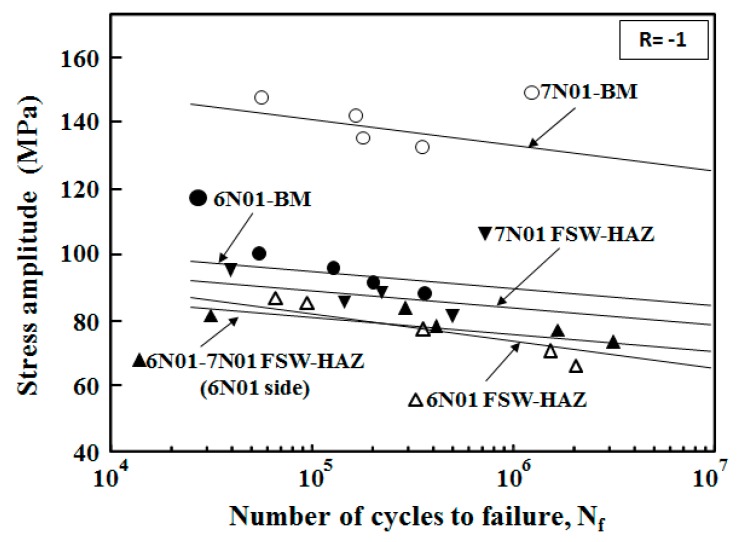
S-N curve for the plate specimens cut from the FSW joints.

**Figure 11:**
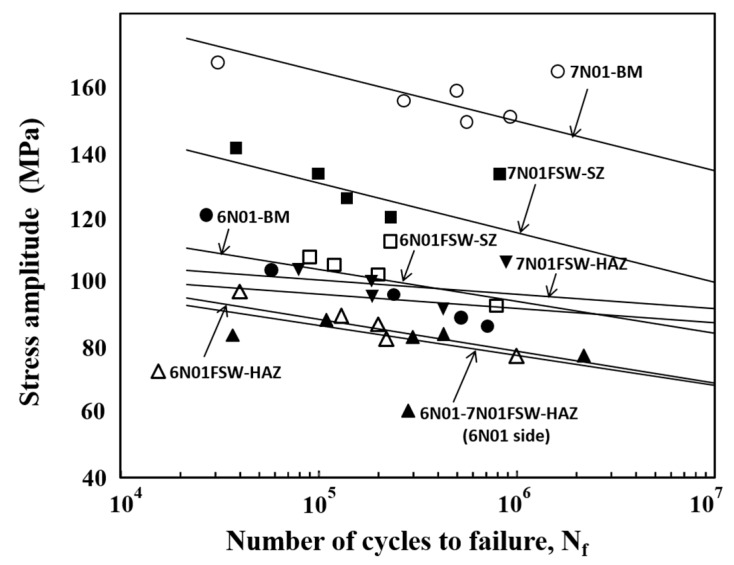
S-N curves for the small round bar specimens cut from the respective locations.

From the fatigue strength tests, S-N curves for the plate specimens of the 7N01 FSW and the 6N01-7N01 FSW are shown in Figure 10. For comparison purposes, those for the 6N01 FSW are also shown in the figure by combining Figure 9, Figure 10 and Figure 11 from [12]. Fatigue strengths *σ_w_* defined as the stress amplitude at 10^7^ cycles and determined from Figure 11 are listed in Table 3. From the careful observation of the fracture location of the FSW plate specimen, the fracture locations of the 7N01 FSW and the 6N01-7N01 FSW were in the HAZ and in the HAZ of the 6N01 side, respectively. It was also found from Figure 10 that the fatigue strength of the 6N01-7N01 FSW was almost the same as that of the 6N01 FSW.

For investigating more details of the fatigue behavior of the FSW joints, fatigue tests of the small round bar specimens were carried out to obtain local fatigue strengths of respective locations of the FSW joints. The results are shown in Figure 11. Fatigue strengths *σ_w_* at 10^7^ cycles determined from Figure 11 are listed in Table 3. For the 7N01 FSW, it was found that the fatigue strength of HAZ was the lowest, that of SZ was the intermediate, and that of BM was the highest. Since no residual stress effect could be assumed, the fatigue fracture of the 7N01 FSW occurred in the HAZ with the lowest fatigue strength. For the 6N01-7N01 FSW, since the fatigue strength of HAZ in the 6N01 side was the lowest compared to the other regions, fatigue fracture of the 6N01-7N01 FSW occurred in the HAZ of the 6N01 side of the joint. The fatigue strength of the HAZ in the 6N01 side was almost the same as that of the HAZ of the 6N01 FSW, as seen in Figure 11, which did not contradict the fatigue test results of the plate specimen, that the fatigue strength of the 6N01-7N01 FSW almost coincided with that of the 6N01 FSW.

## 4. Fatigue Strength Estimation Method

From the foregoing fatigue test results, it is found that the fatigue fracture of the aluminum alloy similar and dissimilar FSW joints occurs at the location of the lowest local fatigue strength, which is also the same as the location of the lowest local tensile strength and the lowest micro-hardness location. Therefore, if explicit relationships among local fatigue strength, local tensile strength, and micro-hardness could be determined with narrow enough scatter, the fatigue strength of the aluminum alloy FSW joint would be predicted based on the micro-hardness measurement without the fatigue and tensile tests. In Section 3.3, the relationship between the tensile strength and hardness has already been obtained as Equation (1) for aluminum alloys. Therefore, if the relationship between fatigue strength and tensile strength could be determined, the equation for predicting fatigue strength from the hardness would be obtained by combining with Equation (1).

### 4.1. Relationship between Fatigue Strength and Micro-Hardness

**Figure 12:**
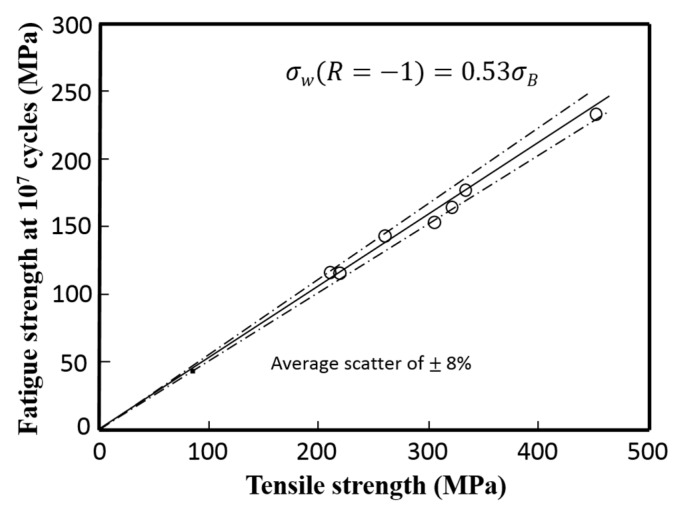
Relationship between the fatigue strength under *R* = −1 at 10^7^ cycles and tensile strength.

The relationship between fatigue strength *σ_w_* at 10^7^ cycles and tensile strength *σ_B_* for the small round bar specimen was investigated. The fatigue strengths listed in Table 3 are under *R* = 0.1. According to the literature, the fatigue strength under *R* = −1 and the S-N curve under *R* = −1 are commonly adopted as the standard data. Therefore, by assuming the modified Goodman’s diagram, *σ*_*w*(*R*)_ = *σ*_*w*(*R* = −1)_ (1 − *σ_m_*/*σ_B_*), the fatigue strength under *R* = 0.1 was reduced to that under *R* = −1, where the value of *σ_m_*/*σ_B_* was assumed to be 0.4 based on the experimental average value, and then the equation *σ*_*w*(*R* = −1)_ = 1.67*σ*_*w*(*R* = 0.1)_ was obtained. The resultant relationship between fatigue strength under *R* = −1 at 10^7^ cycles and tensile strength is shown in Figure 12. From the figure, the relationship between *σ*_*w*(*R* = −1)_ and *σ_B_* can be approximated as:

(3) *σ*_*w*(*R* = −1)_ = 0.53*σ_B_*

The average percentage of scatter S was 4.0%. This relationship between fatigue strength and tensile strength is almost similar to those of extruded bulk aluminum alloys reported by Shikama et al. [23] and also that proposed for aluminum alloys by Forrest [24]. Since each specific region of the FSW joint follows the same relationship for the extruded or rolled bulk materials, no special microstructural effect to degrade fatigue strength is speculated to occur in the present FSW joint, where welding defects could hardly be found and the residual stress effect was negligible.

By combining Equation (1) for tensile strength vs. hardness and Equation (3) for fatigue strength vs. tensile strength, the relationship between fatigue strength and hardness can also be obtained as:

(4) *σ*_*a*(*R* = −1)_ = 1.68 HV.

If the stress ratio is R = 0.1 as in the present experiment, the similar relationship is given as:

(5) *σ*_*a*(*R* = 0.1)_ = 1.01 HV.

**Figure 13:**
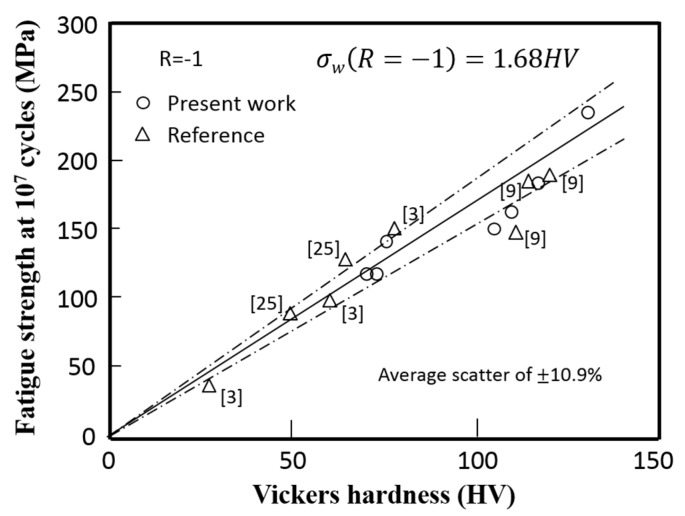
Relationship between the fatigue strength under *R* = −1 at 10^7^ cycles and hardness.

If this relationship will be effective in a wide range of FSW joints of aluminum alloys, it may be possible to estimate the fatigue strength of the FSW joint from the hardness measurements, which will be greatly useful for fatigue design of the FSW joint to improve the cost performance. To confirm the accuracy of estimation of the fatigue strength of the FSW joint from the hardness measurements, the relationship between fatigue strength and hardness for the present experiments are plotted in Figure 13. The available data points from the references with the fatigue strength under *R* = −1 and the hardness distribution are also plotted in the figure. It is found from the figure that Equation (4) holds for a wide range of aluminum alloys with an average percentage of scatter of 10.9%. It should be noted that if the residual stress is induced due to the geometry of the components or constraint by a combination of components, the effect of residual stress should be taken into account for the local fatigue strength in the FSW joints.

### 4.2. Confirmation of the Predicted Fatigue Strength

**Figure 14:**
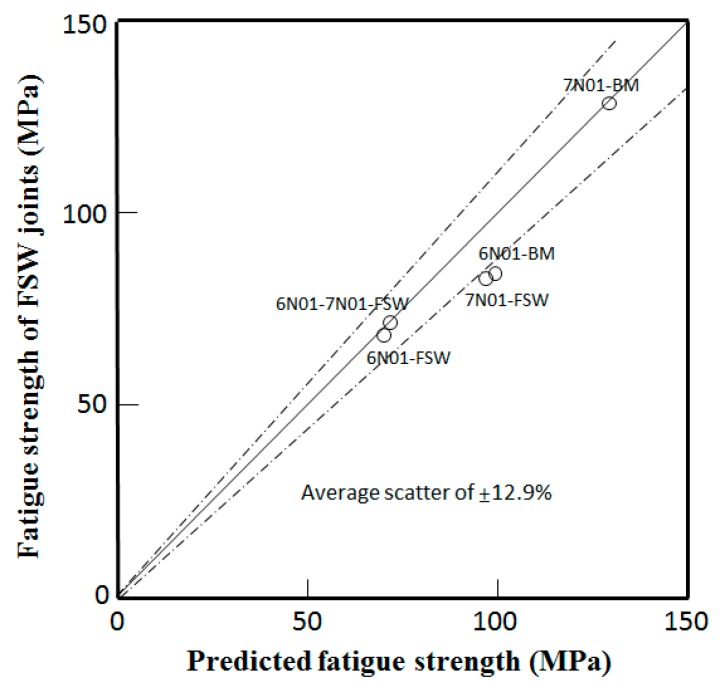
Relationship between fatigue strength predicted by Equation (5) and the experimental fatigue strength of FSW joints under *R* = 0.1.

The relationship between the fatigue strength predicted by Equation (5) and the experimental fatigue strength of the FSW joints under a stress ratio of 0.1 is shown in Figure 14. It is found from the figure that the predicted fatigue strength of the FSW joints are in good agreement with the experimental fatigue strength with an average percentage of scatter of 12.9%.

## 5. Conclusions

The tensile strength and fatigue strength of the 7N01 FSW and the 6N01-7N01 FSW were investigated in addition to the previously reported 6N01 FSW. The relationship between the fatigue strength and hardness for the aluminum alloys has been determined for estimating the fatigue strength of aluminum alloy FSW joints from micro-hardness measurements. The main conclusions obtained are as follows:

(1) The microstructural morphology and micro-hardness distribution of the 6N01-7N01 FSW were found to be the same as for the 6N01 FSW in the 6N01 side of the 6N01-7N01 FSW and for the 7N01 FSW joint in the 7N01 side of the 6N01-7N01 FSW.
(2) In the stir zone of the 6N01-7N01 FSW, both the 6N01 and 7N01 were recrystallized but the two materials were isolated and not homogenized due to an un-melting process of the FSW.
(3) The lowest hardness and tensile strength were found at the HAZ for all three kinds of FSW joints.
(4) Fatigue fracture also occurred at the HAZ with the lowest hardness and tensile strength: For the 6N01-7N01 FSW, fatigue fracture occurred at the HAZ in the 6N01 side, where the hardness and local tensile strength were the lowest.
(5) Based on the present experimental results, the relationships between the tensile strength and hardness, and between the fatigue strength and hardness were approximated as:
  *σ_B_* = 3.05 HV
  *σ*_*w*(*R* = −1)_ = 1.68 HV and *σ*_*w*(*R* = 0.1)_ = 1.01 HV
  These relationships will be useful and will offer a cost-performance method for fatigue design of aluminum alloy FSW joints, to estimate the tensile strength and fatigue strength of FSW joints from the micro-hardness measurements.

## Figures and Tables

**Table 1:** Chemical composition of the materials used (mol %).

Alloy	Mg	Si	Zn	Cu	Mn	Cr	Zr	Ti	Fe	Al
6N01	0.60	0.65	0.25	-	0.50	0.30	-	0.10	0.35	Bl
7N01	1.71	0.12	4.50	0.15	0.35	0.16	0.15	0.05	0.21	Bl

**Table 2:** Tensile strength of the friction stir welding (FSW) joints.

FSW joint	Plate Specimen		Small Round Bar Specimen	
	Tensile Strength (N/mm^2^)		Tensile Strength (N/mm^2^)	
6N01	base metal (BM)	285	BM	305
	FSW (fractured at HAZ)	185	stir zone (SZ)	262
			heat affected zone (HAZ)	218
7N01	BM	431	BM	450
	FSW (fractured at SZ)	308	SZ	328
			HAZ	322
6N01-7N01	FSW (fractured at HAZ in 6N01 side)	176	SZ (in 7N01 side)	326
			HAZ (in 6N01 side)	215

**Table 3:** Fatigue strength *σ_w_* at 10^7^ cycles under *R* = 0.1 of the FSW joints.

FSW Joint	Plate Specimen Fatigue Strength (N/mm^2^)		Small Round Bar Specimen Fatigue Strength (N/mm^2^)	
6N01	BM	86	BM	88
	FSW (fractured at HAZ)	65	SZ	86
			HAZ	68
7N01	BM	126	BM	140
	FSW (fractured at SZ)	81	SZ	101
			HAZ	95
6N01-7N01	FSW (fractured at HAZ in 6N01 side)	72	HAZ (in 6N01 side)	70
